# The Improvement of Hypertension by Probiotics: Effects on Cholesterol, Diabetes, Renin, and Phytoestrogens

**DOI:** 10.3390/ijms10093755

**Published:** 2009-08-27

**Authors:** Huey-Shi Lye, Chiu-Yin Kuan, Joo-Ann Ewe, Wai-Yee Fung, Min-Tze Liong

**Affiliations:** School of Industrial Technology, Universiti Sains Malaysia, 11800 Penang, Malaysia

**Keywords:** probiotic, antihypertension, cholesterol, diabetes, renin, phytoestrogen

## Abstract

Probiotics are live organisms that are primarily used to improve gastrointestinal disorders such as diarrhea, irritable bowel syndrome, constipation, lactose intolerance, and to inhibit the excessive proliferation of pathogenic intestinal bacteria. However, recent studies have suggested that probiotics could have beneficial effects beyond gastrointestinal health, as they were found to improve certain metabolic disorders such as hypertension. Hypertension is caused by various factors and the predominant causes include an increase in cholesterol levels, incidence of diabetes, inconsistent modulation of renin and imbalanced sexual hormones. This review discusses the antihypertensive roles of probiotics via the improvement and/or treatment of lipid profiles, modulation of insulin resistance and sensitivity, the modulation of renin levels and also the conversion of bioactive phytoestrogens as an alternative replacement of sexual hormones such as estrogen and progesterone.

## 1. Introduction

Probiotics are viable microorganisms that confer health benefits to the host once consumed in adequate amounts, primarily by promoting the proliferation of beneficial gastrointestinal indigenous microflora. Various microorganisms have been found to posses such properties, although *Lactobacillus* and *Bifidobacterium* are the most common probiotic bacteria used as food adjuvants. A number of gastrointestinal health benefits have been reported upon consumption of probiotic organisms, including the alleviation of diarrhea, improvement of irritable bowel syndrome, lactose intolerance and antibacterial properties.

The National Heart, Lung, and Blood Institute has classified hypertension for adults (aged 18 years or above) into four main categories. Normal blood pressure (BP) is defined as a systolic BP (SBP) of less than 120 mm Hg and a diastolic BP (DBP) of less than 80 mm Hg, while prehypertension has been defined as SBP of 120–139 mm Hg and DBP of 80–90 mm Hg. Those who are at the risk of stage one progression hypertension are defined as those with SBP of 140–159 mm Hg and DBP of 90–99 mm Hg, while stage two includes those with SBP of above 160 mm Hg and DBP above 100 mm Hg. The new guidelines of blood pressure are the decreased version of the previously accepted blood pressure range in order to encourage more proactive and earlier treatment of high blood pressure. This is because the risk of heart disease and stroke increases at blood pressures above SBP/DBP values of 115/75 mm Hg [1].

Hypertension may be either primary or secondary. Primary hypertension is diagnosed with no known cause and accounts for 95% of all hypertensive cases [1], while secondary hypertension may result from pregnancy, diseases such as sleep apnea, Cushing’s syndrome, kidney malfunction, and as a side-effect of various drugs. Although the exact causes of primary hypertension remain unclear, several factors that increase the risks of primary hypertension have been identified: hypercholesterolemia, diabetes, increased physiological production of renin and an imbalanced sexual hormones profile.

Hypercholesterolemia and obesity are strongly associated with primary hypertension. The over-activation of the sympathetic nervous system by the action of leptin could alter lipid profiles and increase blood pressure by causing peripheral vasoconstriction and by increasing renal tubular sodium reabsorption [2]. Insulin resistance has also been associated with impaired endothelium-dependent vasodilatation which could contribute to increased blood pressure [3]. Insulin resistance could raise blood pressure, either by preventing the vasodilatory effects of the hormone or, via the attendant hyperinsulinemia, by upregulating the sympathetic and the antinatriuretic tone [4]. Primary hypertension has also been associated with renin, an acid proteinase generated from the inactive precursor prorenin by the action of kallikrein [5]. It is released whenever depletion of salt or stimulation of *β*_2_-receptors by aldosterone occurs. Renin plays a role in the renin-angiotensin system by hydrolyzing angiotensinogen to yield the inactive angiotensin I. Angiotensin I is further converted into angiotensin II by angiotensin-converting enzyme. Angiotensin II causes vasodilation and induces the release of aldosterone and therefore increases sodium concentration and elevates blood pressure. Additionally, an imbalanced profile of hormones such as estrogens, progesterone and aldosterone has also been found to induce hypertension.

Although the use of probiotics has been primarily associated with the improvement of gastrointestinal health, recent evidence has also shown that probiotics play an important role in other metabolic diseases leading to antihypertensive effects. Thus, this review highlights and discusses the roles of probiotics on the modulation of lipid profiles, insulin, renin and sexual hormones in the effort to reduce hypertension.

## 2. Hypocholesterolemia and Antihypertension

The prevalence of increased total blood cholesterol in the developed and developing nations remains high and has increased in adults, children and adolescents. Hypertension is often associated with hypercholesterolemia or lipid abnormality and obesity [6]. Patients with hypertension also frequently have low levels of high-density lipoprotein (HDL) cholesterol and higher levels of triglycerides. In other words, hypertension occurs more frequently for hypercholesterolemic subjects, as compared to normolipid men and women. The elevation of blood pressure has been found to be greatly induced when total cholesterol level exceeds 6.4 mmol/L. This may increase cardiac output and peripheral vascular resistance that causes an elevated blood pressure. Therefore, lipid metabolism disorders are often the causes of hypertension. A variety of past *in vitro* experiments and *in vivo* trials have provided experimental evidence to support the roles of probiotics in lowering serum cholesterol and improving lipid profiles, which subsequently leads to a reduced risk of hypertension.

Mann and Spoerry [7] were the first researchers to illustrate the hypocholesterolemic effect of wild *Lactobacillus*-fermented milk. Emerging evidence has indicated that lactobacilli are not the only ones that exhibit hypocholesterolemic effects, but bifidobacteria could also cause a significant reduction in serum cholesterol when cholesterol is elevated. This is because cholesterol synthesis and absorption mainly occurs in the intestines, therefore intestinal microflora have profound effects on lipid metabolism. Past studies have demonstrated that probiotics could improve lipid disorders such as lowering blood cholesterol levels and increasing resistance of low-density lipoprotein to oxidation, thus leading to a reduced blood pressure [8].

Kieling *et al.* [9] used a randomized, crossover, and placebo-controlled design trial involving 29 women to evaluate the hypocholesterolemic effect of yoghurt supplemented with *L. acidophilus* 145 and *B. longum* 913. The crossover study, of 21 weeks’ duration, involved the administration of 300 g/day yoghurt, and the results obtained showed that HDL-cholesterol was increased significantly (P < 0.05) by 0.3 mmol/L and the ratio of LDL/HDL cholesterol decreased from 3.24 to 2.48. Sindhu and Khetarpaul [10] conducted another placebo-controlled study to evaluate the effects of a probiotic fermented food on serum cholesterol levels in 20 young Swiss mice. The experimental group was fed a food mixture containing probiotics and 1% cholesterol while the control group was fed food without probiotics, but containing 1% cholesterol for 42 days. The authors reported that the feeding of *L. casei* NCDC-19 (10^9^ CFU) and *Saccharomyces boulardii* (10^9^ CFU) caused a 19% reduction in the total serum cholesterol, while LDL cholesterol levels was reduced by 37% after the 42 day feeding trial. In another study, De Rodas *et al.* [11] used a placebo-controlled design trial that involved 33 hypercholesterolemia-induced pigs (Yorkshire barrows) to examine the hypocholesterolemic effect of probiotic. The authors reported that pigs fed with *L. acidophilus* ATCC 43121 (2.5 × 10^11^ cells per feeding) for 15 days showed a reduced total blood cholesterol by 11.8% compared to the control that was not fed the probiotic. Park *et al.* [12] also evaluated the effects of probiotic on cholesterol metabolism in 36 male Sprague-Dawley hypercholesterolemic rats. The authors found that the supplementation of *L. acidophilus* ATCC 43121 (2 × 10^6^ CFU/day) for 21 days not only reduced total serum cholesterol by 25%, but also significantly (P < 0.05) reduced very low density lipoprotein, intermediate density lipoprotein and LDL cholesterol, compared to the control.

Several mechanisms of cholesterol reduction by probiotics via control of cholesterol metabolism have been proposed. One of these proposed mechanisms is the removal of cholesterol by assimilation. The assimilation of cholesterol by probiotics in the small intestine could reduce serum cholesterol by reducing cholesterol absorption in the intestines [13]. Probiotics must be viable and growing, in order to be able to remove or assimilate cholesterol [14]. In an *in vitro* study, Tahri *et al.* [15] reported that growing cells of *Bifidobacterium* sp. were able to remove cholesterol from a broth containing bile salt through assimilation of cholesterol. In addition, Aloglu and Oner [16] showed evidence that probiotic bacteria not only assimilated cholesterol in aqueous media, but also in semisolid media such as cream and butter.

Using *in vitro* experiments, Liong and Shah [14] reported that cholesterol could be removed from media by *L. acidophilus* not only through assimilation during growth, but also through binding of cholesterol to the cellular surface. This mechanism was proposed when both non-growing cells and dead cells were also found to remove cholesterol. Non-growing and dead-cells of *Lactococcus. lactis* subsp. *lactis* bv. *diacetylactis* N7 were also found to remove cholesterol *in vitro* via binding of cholesterol to cellular surface [17]. Tahri *et al.* [15] have previously reported that cholesterol could be attached strongly to the cellular surface, where more than 40% of cholesterol was extracted from cells of *B. breve* ATCC 15700 only after several washings and sonications. In addition, past studies have reported that some probiotics could produce exopolysaccharides (EPS) which adhered to the cell surface and could absorb cholesterol. Kimoto-Nira *et al.* [17] previously suggested cholesterol was bound to bacterial cells and this was a result of the chemical and structural properties of their cell wall peptidoglycans, which contain various amino acid compositions that facilitate the attachment of cholesterol to cellular surfaces.

Other researchers have suggested that the incorporation of cholesterol into cellular membranes could be another mechanism to reduce cholesterol in media. Razin [18] found that most of the cholesterol from the medium was incorporated into the cytoplasmic membrane, however the outer membranes of the intact cells are more easily accessed by cholesterol. There was twice the amount of cholesterol in the protoplast membranes than that of intact cells. This indicated cholesterol could preferentially bind to the cytoplasmic membranes [19]. In a previous study, Noh *et al.* [20] found that cholesterol uptake by *Lactobacillus acidophilus* ATCC 43121 occurred during growth. However, most assimilated cholesterol recovered from the cells was not metabolically degraded. Therefore, the authors suggested that the removal of cholesterol may also due to the ability of *L. acidophilus* ATCC 43121 to incorporate cholesterol into cellular membranes during growth. In addition, Liong and Shah *et al.* [14] reported differences in fatty acid distribution patterns for cells grown with or without cholesterol. The authors found that lower amount of total saturated fatty acids and higher amount of total unsaturated fatty acids were recovered from cells grown in medium containing cholesterol compared to those in the absence of cholesterol. This was attributed to the incorporation of cholesterol into the membrane rather than cellular synthesis because lactic acid bacteria living under fatty conditions might lose their ability to synthesize lipids or fatty acids [21]. Cholesterol that is incorporated into bacterial cells during growth in the small intestine is less absorbed into the enterohepatic circulation, thus could lead to reduced serum cholesterol in humans.

**Figure 1.**
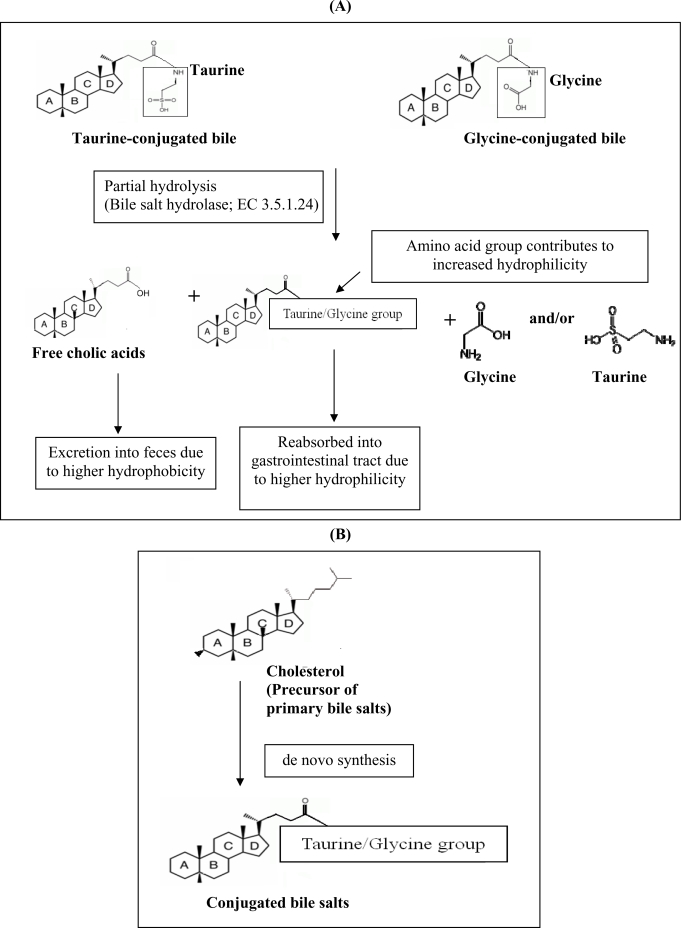
Postulated mechanism of BSH on bile (A) and the role of cholesterol as the precursor for synthesis of new bile acids (B).

Another hypocholesterolemic mechanism that was postulated involves the ability of certain probiotics to enzymatically deconjugate bile acids. Deconjugation of conjugated bile salts to deconjugated bile salts is catalyzed by bile salt hydrolase (BSH; cholyglycine hydrolase; EC 3.5.1.24), which is the enzyme that catalyzes the hydrolysis of glycine- and/or taurine-conjugated bile salts into amino acid residues and free bile acids [22]. Deconjugation of bile salts mainly occurs in the small and large intestines of mammalian hosts. However, the exact location of this metabolite activity is dependent on the distribution of the host species. For example, bile salt deconjugation starts in the small intestine of mice, whereas in humans significant deconjugation begins at the end of the ileum and is completed in the large bowel [23]. BSH activity has been detected in intestinal bacteria such as *Lactobacillus* and *Bifidobacterium* sp. [22]. It has been demonstrated previously that the removal of cholesterol by *L. reuteri* CRL 1098 was closely related to the BSH activity of the cells, which hydrolysed the amide bond of bile salts releasing the corresponding free bile acids [24]. The mechanism of actions of BSH on bile is shown in Figure 1a. Conjugated bile salts are readily absorbed into the gastrointestinal tract due to higher hydrophilicity, while free bile acids are less soluble and thus less efficiently reabsorbed into the intestines, compared to conjugated bile salts, and thus are more prone to be excreted with the feces. This will increase the need for the synthesis of new bile acids to replace the lost ones. Since cholesterol is the precursor for the *de novo* synthesis of new bile acids (Figure 1b), the use of cholesterol to synthesize new bile would lead to a decreased concentration of cholesterol in blood.

## 3. Roles of Probiotics on Diabetes

Diabetes and hypertension are co-morbidity diseases that frequently occur together in the same patients. In a large prospective cohort study conducted by Gress *et al.* [25] that involved 12,550 adults, the development of type II diabetes was almost 2.5 times more likely in those with hypertension than in their normotensive counterparts. Similarly, recent data suggested that hypertension is approximately twice as frequent in patients with diabetes compared with patients without the disease [26]. The occurrence of hypertension tends to be relatively lower in patients with type I diabetes and affects 30% of type 1 diabetes patients [27].

There is a complex relationship between insulin resistance, diabetes and essential hypertension. Insulin resistance is a phenomenon whereby body tissues, namely skeletal muscle, adipose tissue and the liver have impaired biological and physiological responses to circulating insulin [28]. Essential hypertension is responsible for 90–95% of patients diagnosed with hypertension with no clear cause [29]. Lind, Bente and Lithell [30] estimated that about 25–47% of hypertensive patients have impaired insulin resistance or impaired glucose tolerance. Sowers, Epstein and Frohlich [26] observed that untreated essential hypertensive individuals have higher fasting and postprandial insulin levels than the normotensive individuals. Therefore, those with essential hypertension are more prone to develop diabetes than normotensive persons.

Both diabetes and high blood pressure are risk factors for the development of macrovascular and microvascular complications. Therefore, rigorous control of blood pressure and glucose are paramount to decrease the morbility and mortality of hypertensive diabetes individuals [31]. A wide range of antihypertensive drugs is available in the market but not all offer beneficial effects in hypertensive diabetes. Therefore, the development of new therapy methods is needed in order to produce an efficient method for preventing or reducing the occurrence of diabetes and hypertension with the least side effects. The consumption of probiotics is a new therapeutic strategy in preventing or delaying the onset of diabetes and subsequently reducing the incident of hypertension.

An induction in insulin resistance often leads to diabetic dyslipidemia, and this is highly increased by high levels of plasma total cholesterol, LDL cholesterol, and very low density lipoprotein (VLDL) cholesterol [28]. The efficacy of probiotics in reducing serum cholesterol levels as demonstrated by various in-vivo models could subsequently improve insulin resistance. It has been suggested that the consumption of probiotics can lower the onset of insulin resistance and consequently reduce the incident of hypertensive conditions that are closely related to diabetes.

**Figure 2.**
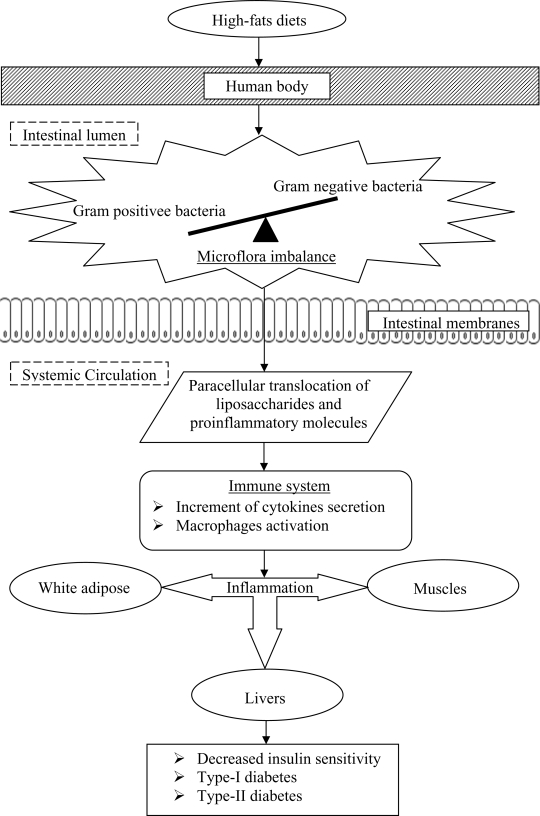
Postulated mechanism involved in the onset of diabetes upon prolonged consumption of high-fat diets.

Past studies have also postulated that the onset of diabetes is associated with a poor inflammatory status of the individuals that have consumed high-fats diets over prolonged periods (Figure 2). Cani *et al.* [33] evaluated the effects of a high-fat diet on lipopolysaccharides using mice as a model. The authors demonstrated that the composition of natural intestinal gut microflora often determine the degree of inflammation contributing to the onset of diabetes and obesity. The concentration of plasma lipopolysaccharides, the proinflammatory factor, is inversely correlated with the population of *Bifidobacterium spp.* It has also been reported that in high-fat diet-induced diabetes, the concentration of *Bifidobacterium spp.* in the gut was positively correlated with improved glucose tolerance and glucose-induced insulin-secretion as well as decreased diabetes endotoxemia, plasma and adipose tissue inflammatory cytokines [33]. Several studies have also shown that bifidobacteria can reduce the intestinal endotoxin levels and improve mucosal barrier thus reducing systemic inflammation and subsequently reduced the incidence of diabetes [34].

In a randomized, double-blind, placebo controlled human study that involved 25 healthy elderly volunteers (median age 69 y; range 60 ± 83 y), the consumption of low-fat milk containing 1.5 × 10^11^ CFU of *Lactobacillus lactis* twice daily for a period of six weeks was found to enhance the immune response of the elderly, without the release of inflammatory cytokines, thus reducing the onset of systemic inflammatory induced diabetes [35]. Another study conducted by Matsuzaki *et al.* [36] had shown significant reduction of plasma glucose (P < 0.05) in 4-week-old non-insulin dependent diabetes mellitus male KK-A^y^ mice that were fed 2 mg of lyophilized *Lactobacillus casei* five times a week for an experimental period of 16 weeks. The KK mice were an inbred strain of Japanese native mice that possessed various diabetic features, while A^y^ was the yellow obese gene. The incorporation of A^y^ gene into KK mice produced insulin resistant models. The authors concluded that the consumption of probiotics inhibited the production of proinflammatory cytokines that would lead to the occurrence of systemic inflammatory induced diabetes, and eventually inhibited the onset of diabetes related hypertension.

The incidence of systemic inflammatory induced diabetes was also found to increase with the decrease ratio of Gram-positive:Gram-negative intestinal microflora. Such a decreased ratio tends to increase the availability of proinflammatory molecules and lipopolysaccharides in the body that are responsible for systemic inflammation, and thus triggers the occurrence of diabetes.

Lipopolysaccharides are the main components found in the outer membrane of Gram negative bacteria. The supplementation of probiotics reduces the population of pathogenic Gram negative bacteria in the gut and modulates immune response. Several mechanisms for the inhibition of Gram negative bacteria by probiotics have been postulated, including the competition for nutrients and adhesion sites, production of direct inhibitory compounds such as bacteriocins, and lowering of colonic pH by the production of short chain fatty acids [37].

Past studies have demonstrated the beneficial effects of co-consumption of probiotics with diabetic drug on controlling diabetes. Gliclazide is an oral anti-diabetic sulfonylurea drug that has beneficial extrapancreatic effects when insulin therapy is insufficient. Al-Salami *et al.* [38] evaluated the effects of probiotics on the uptake of gliclazide by using diabetic and healthy Wistar rats (n = 10). These rats were fed probiotics (75 mg/kg body weight) for three days, after which a gliclazide suspension (20 mg/kg) was administered. The authors reported that a two-fold probiotics-induced increment (p < 0.01) in gliclazide uptake was observed in the diabetic rats that resulted in a reduction of blood glucose levels by two-fold via insulin-independent mechanisms. Such findings indicate the beneficial effects of probiotics in treating diabetes in synergism with other diabetes drug and thereby reduced the incidence of diabetes related hypertension.

It has been found that high fructose diets induce type II diabetes that is associated with insulin resistance, hyperinsulinemia, hypertriglyceridemia and hypertension [28]. This is caused by the mobilization and accumulation of fructose in the liver that increases the rate of lipogenesis and synthesis of triacylglycerol. The catabolism of fructose ultimately induces insulin resistance [39]. In a study conducted by Yadav, Jain and Sinha [28], it was found that the administration of dahi (an Indian fermented milk product) containing *Lactobacillus acidophilus, L. casei* and *L. lactis* to high fructose-induced diabetic rats for eight weeks decreased the accumulation of glycogen in the liver of rats compared to the control that was not fed the probiotics.

The authors also found that plasma total cholesterol, LDL cholesterol and VLDL cholesterol levels were reduced, leading to a decrease in the incidence insulin resistance. The mechanisms proposed are inhibition of insulin depletion, preservation of diabetic dyslipidaemia and inhibition of lipid peroxidation and nitrite formation in the rats. This finding indicated that the consumption of probiotic products reduced the risk of diabetes and diabetic-linked-complications of individuals with fructose induced insulin resistance.

The sensitivity of insulin in the regulation of blood glucose and fat metabolism is decreased by the dysfunction of pancreatic *β*-cells [40]. Alloxan is a type of toxic glucose analogue that selectively destroys insulin-producing pancreatic *β*-cells upon consumption, leading to the development of insulin-dependent diabetes mellitus. The mechanism is thought to be initiated by the production of free radicals from reactive oxygen species (ROS) formed by alloxan that preferentially accumulates in *β*-cells. Matsuzaki *et al.* [41] reported an inhibition of alloxan-induced disappearance of pancreatic *β*-cells in a group of 7-week-old alloxan-induced diabetic-BALB/c mice that were orally fed a diet containing lyophilized *Lactobacillus casei.* The authors reported that the administration of the probiotic reduced the occurrence of insulin deficiency that is associated with hyperglycemia. Similarly, in another study, Matsuzaki *et al.* [42] observed that the inhibition of autoimmune destruction of pancreatic *β*-cells was decreased upon oral feeding of a diet containing 0.05% heat-killed lyophilized *L. casei* in 4-week-old female non-obese insulin dependent diabetes (NOD) rats. Thus, the consumption of probiotics confer protections to the well being of pancreatic *β*-cells that plays a significant role in the production of insulin molecules and prevents the onset of insulin dependent diabetes as well as diabetes-related hypertension.

Other *in-vivo* studies have also demonstrated the roles of probiotics on glucose tolerance and insulin resistance. Tabuchi *et al.* [43] conducted a study investigating the effects of *Lactobacillus rhamnosus* GG (GG) on blood glucose levels and glucose tolerance levels in neonatally streptozotocin-induced diabetes rats. The diabetic and normal rats were weaned at four weeks after birth and fed a diet containing 6% lyophilized GG cells and normal diet, respectively. Each group was given 20 g of their respective diet daily for nine consecutive weeks. The authors reported a delay in the elevation of glucose intolerance and hyperglycemia in neonatal streptozotocin-induced diabetes rats upon oral feeding of the GG cells.

A combination of probiotics strains was also found to be advantageous in reducing the onset of insulin resistance and diabetes in animals. VSL#3 is a commercially available mixture of probiotics containing a high-concentration (450 billion colonies/sachet) of viable, lyophilized *bifidobacteria, lactobacilli*, and *Streptococcus thermophilus*. Li *et al.* [44] conducted a study involving 48 ob/ob mice that were fed a diet containing aliquots of VSL#3 bacteria (1.5 × 10^9^ colonies/mouse/day). Ob/ob mice are ob-gene modified mice that produce excessive leptin hormone which is important in the control of appetite in the mice leading to the development of obesity due to excessive eating. The authors found that the administration of VSL#3 probiotics mixture improved hepatic insulin resistance in diabetic mice, after 4 weeks of treatment as compared to the control.

The roles of probiotics in reducing the onset of insulin resistance, hyperglycemia and diabetes dyslipidemia have yielded positive findings. It is believed that probiotics could be used as an alternative preventive measure and treatment for diabetes and subsequently reduces the risks of diabetic-associated hypertension.

## 4. Regulation of Renin

Blood pressure is controlled by a number of different interacting biochemical pathways. Typically, the regulation of blood pressure has been associated with renin-angiotensin system (RAS) which involved angiotensin-converting enzyme (ACE) [1]. RAS regulates blood pressure, fluid and electrolyte balance. Renin is an acid proteinase generated from the inactive precursor pro-renin by the action of kallikrein [5]. It is released whenever depletion of salt or stimulation of *β*_2_-receptors by aldosterone occurs. In RAS, renin hydrolyses plasma angiotensinogen, thus liberating the inactive angiotensin I. The potent vasoconstrictor, angiotensin II is converted from angiotensin I by ACE. Inhibition of renin activity may be achieved as a result of angiotensin II production [1]. Angiotensin II can cause vasoconstriction and induces release of aldosterone and therefore increases sodium concentration and elevates blood pressure. On the other hand, ACE also contributes to the elevation of blood pressure by inactivating the vasodilator brandykinin [45]. Hence, the levels of both angiotensin II and brandykinin allow for the regulation of blood pressure are mainly dictated by the ACE in RAS.

ACE inhibition is a key clinical target for blood pressure control, whereby ACE inhibitors can lower blood pressure by reducing the production of angiotensin II and inhibit the degradation of brandykinin [45]. The ACE inhibitory peptides are inactive within the sequence of the parent protein but can be released by microbial activity [46]. Hence, fermentation is considered to be an effective way to produce the bioactive peptides. ACE inhibitory peptides can be derived from a variety of fermented products including cheese, fermented milk, soymilk and yogurt upon fermentation by various starter microorganisms [47].

In addition to yogurt bacteria and cheese starter bacteria, probiotic bacteria have been demonstrated to produce different bioactive peptides in milk during fermentation [46]. Probiotics are able to grow in milk products because they posses a proteolytic system that degrades casein along with lactose hydrolyzing enzymes [45]. Upon fermentation, the proteinases of various probiotics are capable of releasing ACE inhibitory peptides and thus a blood-pressure lowering effect can be derived from the milk proteins [48]. Several studies have demonstrated that *Lactobacillus helveticus* are capable of releasing antihypertensive peptides which are ACE inhibitory tripeptides Val-Pro-Pro (VPP) and Ile-Pro-Pro (IPP) from milk protein casein [46].

To date, the ability of probiotics in reducing blood pressure has been elucidated through fermentation of food products in order to release bioactive peptides, such as the ACE inhibitory peptides that play a crucial role in RAS. Therefore, antihypertensive effects can be achieved via consumption of dairy adjunct with probiotics. Evidence from *in vitro* and *in vivo* studies has demonstrated the effects of probiotics on hypertension. A study was performed by Donkor *et al.* [49] on the proteolytic activity of several dairy lactic acid bacteria cultures and probiotics as determinants of growth and *in vitro* ACE inhibitory activity in milk fermented with these single cultures. The authors reported that both *Bifidobacterium longum* and *Lactobacillus acidophilus* strains showed ACE inhibitory activity during growth. This was also supported by Ong and Shah [50], who examined the released of ACE inhibitory peptides in Cheddar cheeses made with starter lactococci and probiotics. The authors observed that cheeses made with the addition of *L. casei* and *L. acidophilus* had higher ACE inhibitory activity than those without any probiotic adjunct after 24 weeks at 4 °C and 8 °C, probably due to increased proteolysis. Moreover, ACE-inhibitory peptides have also been found in yogurt, cheese and milk fermented with *L. casei* ssp. *rhamnosus*, *L. acidophilus* and bifidobacteria strains [51]. Recent research studies have shown that soy peptides with inhibitory activity against ACE could be produced by fermentation with probiotics. In a study performed by Ng *et al.* [52] to investigate the growth characteristics and bioactivity of probiotics in a tofu-based medium, both *L. fermentum* and *L. bulgaricus* strains exhibited varying proteolytic activity leading to the production of bioactive peptides with ACE inhibitory activity. Additionally, Fung [53], who evaluated growth characteristics of *L. acidophilus* in soy whey, postulated that proteolytic activity of probiotic gave rise to ACE inhibitory activity in the media. The authors also found that there was a strong correlation between the ACE inhibitory activity and growth of the probiotics. An increased growth has been associated with an increase in the *in vitro* ACE inhibitory activity.

Experimental evidence involving *in vivo* trials has also exhibited positive results. During a 12 week feeding trial on 30 spontaneously hypertensive rats (SHR), a reduction of 17 mm Hg in systolic blood pressure (SBP) was reported upon consumption of sour milk containing 2.5–3.5 mg/kg/day of Val-Pro-Pro and Ile-Pro-Pro fermented by *L. helveticus* LBK-16H (10%) [54]. In another study, Hata *et al.* [55] used a single-blind, placebo-controlled study involving 30 elderly hypertensive patients. The authors reported that ingestion of 95 mL of sour milk fermented with *L. helveticus* and *Saccharomyces cerevisiae* per day for eight weeks had significantly decreased SBP and diastolic blood pressure (DBP) by 14.1 mm Hg and 6.9 mm Hg, respectively. In one of the largest studies, involving 94 hypertensive subjects in a double-blind, placebo-controlled, randomized trial, Jauhiainen *et al.* [56] found that the consumption of 150 mL milk fermented by *L. helveticus* twice a day for 10 weeks could decrease SBP and DBP by 4.1 mm Hg and 1.8 mm Hg, respectively. More studies exhibiting positive effects are presented in Table 1.

To this end, microbial fermentation provides a natural technology applicable for the production of bioactive peptides either from dairy or plant proteins. Consumption of probiotic fermented products not only supplies bioactive peptides but also live probiotic cultures that could confer antihypertensive properties.

## 5. Phytoestrogen and Probiotics

Hypertension has been associated with imbalanced sexual hormones due to strong epidemiological evidences of gender differences in physiological control mechanisms of blood pressure. The prevalence of hypertension often shifts towards postmenopausal women, who have higher arterial blood pressures attributed to the lost of protective effects of ovarian hormones that regulate blood pressure [62]. The sexual dimorphism of blood pressure and development of hypertension seems to have manifested from protective effects of estrogen in women and aggravation by androgen, in addition to the lack of vital estrogen protection, in men. Estrogen and progesterone serve as antihypertensive sex hormones, antagonizing the pro-hypertensive effects of testosterone, exerted in multifaceted mechanisms with direct effects on the vascular, renal and heart cells; or even via indirect effects mediated by humoral factors [62].

The role of estrogen in positive mediation of hypertension is well documented in both animal and human studies. Hence, hormone replacement therapy is often prescribed to post-menopausal women or hypertensive men to alleviate the condition. However, it was noted that estrogen replacement therapy did not result in significant sustained reductions in blood pressure in women who have experienced surgical menopause [63]. There is a natural alternative to estrogen-based hypertension mediation using food-derived phytoestrogens which are enhanced with probiotics to increase its potency.

**Figure 3.**
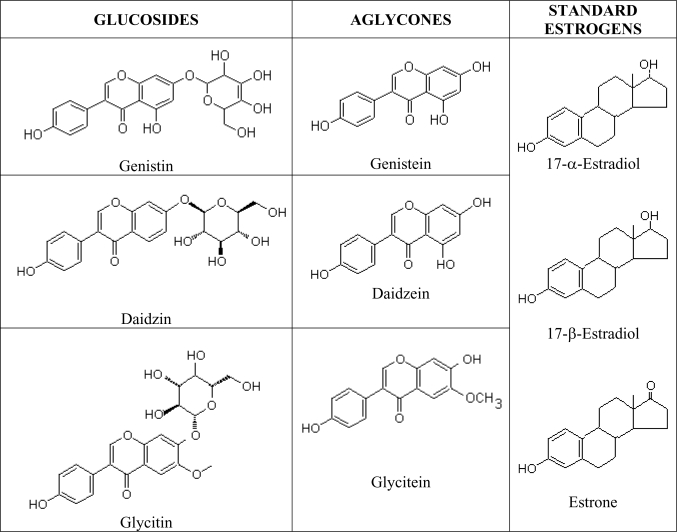
Chemical structures of native glycosides, activated aglycones and standard estrogen.

Phytoestrogens are natural estrogen mimics which possess several common features to the estradiol, including a phenolic ring which is a pre-requisite for binding to the estrogen receptor [64]. Due to their structural similarities to mammalian estrogen [65], soy isoflavanoids may interact with the estrogen activity pathways in the body and induce similar responses in vascular functions as estradiol [66]. The estrogenic properties of soy isoflavones are well known and it was noted as being used by over 30% of women in the US as a supplement or as an alternative to traditional hormone replacement therapy [67]. Isoflavones are isoflavonoids with similar chemical structures as the mammalian estrogens, the estradiols and estrone (Figure 3). This structural similarity have contributed to the protective potential of isoflavones against hormone-dependant diseases, which include menopausal symptoms and cardiovascular disease [68] and they are most commonly found in legumes, such as soy. Administration of intact soy protein containing isoflavones was shown to result in significant vasodilatory effect in post-menopausal women [69].

Gut microflora or probiotics readily hydrolyze the main isoflavonoid glucosides in legumes, such as soy, including genistein and daidzein, into bioactive aglycones [66]. Isoflavone aglycones are absorbed faster and in greater amounts, because aglycones have greater hydrophobicity and a smaller molecular weight compared to the native glucosides [68]. Isoflavones in soy protein are ingested in modest amounts, absorbed following biotransformation by intestinal microflora, and undergo enterohepatic recycling, reaching circulating concentrations that exceed by several orders of magnitude the amounts of endogenous estrogens [64], despite having weaker binding affinity for estrogen receptors [70]. Hence, although isoflavone genistein has a relatively low potency, of one third that of estradiol, high concentrations in plasma may be sufficient to cause a variety of physiological effects [66]. Studies have recorded increases in total circulating estradiol concentrations following soy isoflavone consumption [70]. The 30–50 mg/day threshold intake of dietary estrogens necessary to achieve a biological effect in humans is readily attainable by the inclusion of modest amounts of soy protein [64]. Kano *et al.* [68] showed that isoflavone aglycones were absorbed faster and in greater amounts than when ingested in the form of a beverage like soymilk. Naturally, the high intake of soy and soymilk in Japanese diet has been benefiting the Japanese, as the plasma of Japanese men reportedly contains as much as 7- to 110-fold the concentration of in phytoestrogens a similarly aged group of Finnish men [71].

Bioavailability of phytoestrogens in the human body is greatly influenced by gut microflora. The ability of intestinal bacteria to metabolize isoflavones increases the bioavailability of isoflavones in the form of aglycones and determines the production of biologically important isoflavone metabolites such as equol [72,68], thus enhancing effects on estrogen metabolism [73]. Probiotics have been reported to posses the ability to biotransform glucosides to aglycones. Owing to possessing β-glucosidase that contributed to the bioconversion of glucosides, probiotics could take over the place of intestinal bacteria in releasing the bioactive aglycone from soy foods. Soymilk fermented with some strains of *Bifidobacterium* was found to contain equol [74]. Equol has greater affinity for the estrogen receptors, hence, greater efficacy compared to its precursor daidzein or other isoflavones in receptor-mediated effects of soy [72]. Such effects include the inhibition of production of contraction factors endothelin-1 via ER receptor-dependant mechanisms, leading to vasodilation and anti-hypertensive effect; and inhibition of estrogen sulfotransferase, leading to in-vitro increase of circulating active estrogens [75]. While only 30–50% of the human population are able to metabolize daidzein to equol [67], the administration of soy isoflavone with probiotics can enhance the effects of isoflavones, by mediating the conversion of daidzein to equol, especially in non-equol producers.

Intestinal *β*-glycosidases often biotransform conjugated glucosides to bioactive aglycones via hydrolytic cleavage. However, probiotics that are capable of producing *β*-glycosidase could also liberate such bioactive properties. Consumption of probiotic strains of *Lactobacillus* and *Bifidobacteria* was shown to increase β-glucosidase in humans [73]. The increase in the cleavage enzymes from the fermentative probiotics increases the bioavailability of aglycones in fermented soymilk, resulting in increased isoflavone absorption efficiency which may then manifest in greater physiological effects of the aglycone-enriched fermented soymilk compared to glucoside-enriched unfermented soymilk [68].

Past studies have shown that the concentrations of isoflavone aglycones in soy food are increased upon fermentation by probiotics. In a study performed by Chien *et al.* [76] on the transformation of isoflavone phytoestrogens during soymilk fermentation, the concentration of isoflavones aglycones (daidzein, glycitein and genistein) has increased 100%, while a reduction of 50%–90% in the concentration of glucoside counterparts upon fermentation by *S. thermophilus* and *B. longum* was observed. In addition, Pham and Shah [77] also found that the level of bioactive aglycones increased from 8% in non-fermented soymilk to approximately 50% due to fermentation by *Bifidobacterium* while the concentration of malonyl-, acetyl- and glucosides isomers decreased considerably, with approximately 50%, 60% and 85% hydrolyzed in soymilk fermented by *B. animalis,* respectively. Therefore, it has been strongly suggested that antihypertensive effects can be achieved through the consumption of isoflavone aglycones enriched probiotic-fermented soy products.

The efficiency of isoflavone absorption has been indicated by urinary excretion of the compound. Co-administration of probiotic *Lactobacillus GG* with soy appeared to reduce the urinary excretion of total and individual isoflavones by 40%, suggesting that the addition of high concentrations of the probiotic (10^12^ cfu) enhanced isoflavone deconjugation and/or blocked isoflavone degradation leading to increased bioavailability and circulating levels of isoflavone, as reflected in reduced excretion [67]. In another study, Kano *et al.* [68] investigated the effects of soymilk-based beverages in twelve healthy volunteers consuming untreated, β-glucosidase–treated and fermented soymilk on serum isoflavones concentrations. The authors found that β-glucosidase–treated soymilk and fermented soymilk increased serum isoflavone concentration in a significantly shorter duration compared to the untreated soymilk. The authors concluded that isoflavone aglycones of soymilk were absorbed faster and in greater amounts than their glucosides counterpart and that the metabolism of isoflavones might be affected by the type of soymilk consumed.

There is strong evidence supporting the positive influence of probiotics on hypertension via the mediation of phytoestrogens. More studies can be conducted to identify the specific probiotics with the nutraceutical property of increasing bioavailability of isoflavones, as it may vary between strains. A natural probiotic-phytoestrogen product may serve as a better alternative to the conventional, radical and synthetic hormone replacement therapy prescribed to post-menopausal women to counter hypertension, among other post-menopausal symptoms, as this treatment has also been associated with side effects such as an increased risk of breast cancer, mood swings and insomnia.

## 6. Conclusions

This review has illustrated the potential of probiotics in mediating hypertension via positive modulation of several different physiological systems, apart from its conventional benefits for gastrointestinal health. Probiotics have exhibited antihypertensive potential via the improvement of lipid profiles, insulin resistance, modulation of renin and the bioconversion of bioactive isoflavones. These positive findings suggested the potential use of dietary alternatives such as probiotics, to alleviate the occurrence of metabolic diseases via a less radical approach compared to drugs or hormone therapy, with milder, if not none, known side effects. Probiotics could also serve as a complementary supplement to enhance the well-being for those already suffering the diseases and taking drugs or hormonal therapy to medicate the condition. Further revelation on the potential of probiotics in future research could lead to a boost in probiotic-fermented food industries–dairy and non–dairy. Nevertheless, more studies are needed to better understand the exact mechanisms, *in vivo* target sites, stability and safety, prior to using probiotics as an antihypertensive alternative treatment.

## Figures and Tables

**Table 1.** *In vivo* studies on the effects of probiotic-fermented milk on blood pressure.

**Product**	**Subjects**	**Study Design**	Effect in blood pressure*	**Reference**
*Lactobacillus helveticus* fermented milk, 150 ml/day for 21 wks	39 mild hypertensive; age 30–62	Randomized, placebo-controlled	**SBP**: −6.7 mm Hg **DBP**: −3.6 mm Hg	[57]
*Lactobacillus acidophilus* and *Streptococcus thermophilus* fermented yogurt, 450 ml/day for 8 wks	70 healthy; age 18–55	Randomized, double-blind, placebo- and compliance-controlled, parallel	**SBP**: −4.4 mm Hg **DBP**: −3.4 mm Hg	[58]
*Lactobacillus helveticus* in tablets containing powdered fermented milk, 6 tablets/day for 4 wks	40 high-normal blood pressure (HN), 40 mild hypertension (MH); age not available	Randomized, double-blind, placebo-controlled	**SBP**- MH: −5.0 mm Hg **DBP**- HN: −11.2 mm Hg	[59]
*Lactobacillus helveticus* fermented milk, 160 g/day for 4 wks	46 borderline hypertensive; age 23–59	Randomized, double-blind, placebo-controlled	**SBP**: −5.2 mm Hg	[60]
*Lactobacillus helveticus* fermented sour milk, 150 ml/day; first period 8–10 wks, washout period 3–4 wks, second period 5–7 wks	60 (first period)/39 (second period) mild hypertension; age not available	Two-cross over trial periods with a washout period in between	First period: **SBP**: −16 mm Hg Second period: **DBP**: −11 mm Hg No significant differences in cross over	[61]

^*^ A negative value shows a reduction from baseline registration values. SBP: Systolic blood pressure. DBP: Diastolic blood pressure.
